# Medical Ethics

**Published:** 1848

**Authors:** 


### ARTICLE II.

### MEDICAL ETHICS.

WE give, in this number of the Journal, the Code of Ethics adopted by the National Medical Association, which was recommended by that body for the adoption of subordinate societies, hoping those of our subscribers who may be engaged in organizing these associations, may secure their adoption, and because every physician in the land, whether connected with a medical society or not, should be governed by the excellent precepts in it laid down.

There is great necessity for a uniformity in the conduct of members of the profession towards each other, which can only be secured by a uniformity in their understanding of what is due from one to another. The origin of many of the unfortunate strifes that arise between physicians, may be traced to a difference of opinion in reference to the proper course of conduct each should pursue, which, by having a uniform standard of ethics, as we trust we will have in the code under consideration, might be avoided, harmony and good fellowship promoted, and the profession advanced in dignity, respectability, and, consequently, in usefulness to society. E.

